# Congenital nasal pyriform aperture stenosis

**DOI:** 10.1016/S1808-8694(15)31319-7

**Published:** 2015-10-20

**Authors:** José V. Tagliarini, Victor Nakajima, Emanuel C. Castilho

**Affiliations:** 1 Assistant Professor, Department of Ophthalmology, Otorhinolaryngology and Head and Neck Surgery, Medical School, Botucatu – UNESP; 2 Otorhinolaryngologist, Department of Ophthalmology, Otorhinolaryngology and Head and Neck Surgery, Medical School, Botucatu – UNESP

**Keywords:** nasal cavity, abnormalities, embryology, surgery

## Abstract

The congenital stenosis of pyriform aperture is an unusual cause of neonatal nasal obstruction. It is due to bony overgrowth of the nasal lateral process of the maxilla. Initially this narrowest part of nasal airway was considered an isolated deformity; subsequently the congenital Stenosis of pyriform aperture was thought to represent a microform of holoprosencephaly. In this report a male neonate had respiratory distress, cyclic cyanosis and apnea after delivery. The patient underwent surgical correction of pyriform stenosis by sublabial access. In the follow up, the patient had good evolution. The report of this deformity shows an important cause of neonatal nasal obstruction and its differential diagnosis with bilateral choanal atresia. Congenital stenosis of nasal pyriform aperture can be surgically corrected when necessary.

## INTRODUCTION

Congenital stenosis of pyriform aperture (CSPA) is a rare pathology that occurs in newborns caused by excessive growth of medial nasal process of maxilla. As a consequence, it may take to neonatal respiratory failure^1^. It was initially reported as an isolated deformity, and later it was considered as the presentation of the minor form of holoprosencephaly. It results from abnormal development of prosencephalus and facial structures^2^. CSPA is important in differential diagnosis of the causes of congenital nasal obstruction.

## LITERATURE REVIEW

Congenital stenosis of pyriform aperture (CSPA) was first described by Brown et al. (1989) as a cause of nasal obstruction that occurred in the newborn. It is caused by excessive growth of medial nasal process of maxilla that leads to narrowing of the bony part of nasal cavity^1^. It was initially considered an isolated deformity, but later it was understood as a presentation of a minor form of holoprosencephaly, resultant from abnormal development of prosencephalus and medial facial structures. This hypothesis is based on the observation that solitary central upper incisor, one of the manifestations to holoprosencephaly, is found in many cases of CSPA^2^. Additional evidences to support this hypothesis are described in twin pregnancy. In two reports, one of the children presented holoprosencephaly and the other monozygotic twin presented CSPA^3^, ^4^. Even though the cause of the pathology is still unknown and case reports are sporadic, presence of incisor manifestation together with the pathology has been described in half of the cases^1,^^2^, ^5^. Some reports described cases associated with endocrine affections, such as growth hormone deficit^5^, thyroid dysgenesis^2^, hypothyroidism and episodes of hypoglycemia and absence of anterior hypophysis with panhypopituitarism^6^, ^7^. Another factor that has been described is close topographic relation of initial ontogenetic stages between primordial neural prosencephaly, adenohypophysis, olfaction organ, and facial ectoderm. Facial ectoderm includes nasal cavity ectoderm. Cells of the neural crest of the same territory (prosencephalic neural crest) give origin to osteoblasts of pyriform aperture skeleton^9^. Nasal development starts during the third week of normal fetal development, when olfaction placode pairs are developed, forming nasal invaginations; they divide the frontonasal process into lateral and medial processes. Medial processes merge forming the primitive nasal septum, pre-maxillary process and central part of the upper lip. Developing maxilla merge with the lateral process to obliterate the nasal-optical sulcus and form the lateral nasal wall and pyriform aperture^10^.

Neonates are preferably nasal breathers and any cause of nasal obstruction may take them to severe consequences. Immediate recognition and appropriate treatment are necessary to prevent asphyxia^1^, ^8^, ^11^. A respiratory pattern of cyclic cyanosis relieved by cry may be reported. These symptoms may also occur in patients with bilateral choana atresia^1^, ^8^, ^11^. Examination of the nose may be very difficult because of the reduced size of nasal fossa^11^. Anterior rhinoscopy shows narrow nasal fossa because of submucous bone protrusion, and fossa lumen may be as small as 2mm^11^.

CT scan is the radiological exam of preference in the diagnosis of CSPA and in confirming normal anatomy of choanae. Axial sections can be obtained from the palate to the roof of the orbit^11^.

## CASE REPORT

**Figure 1:**
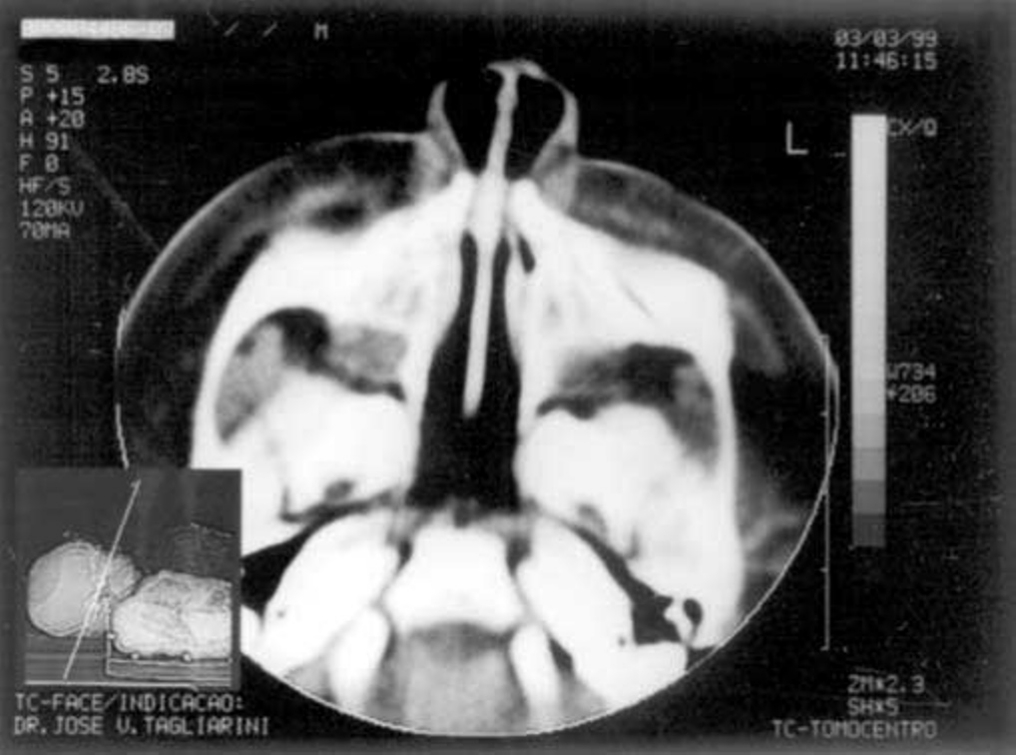
Axial section showing nasal fossa stenosis.

**Figure 2:**
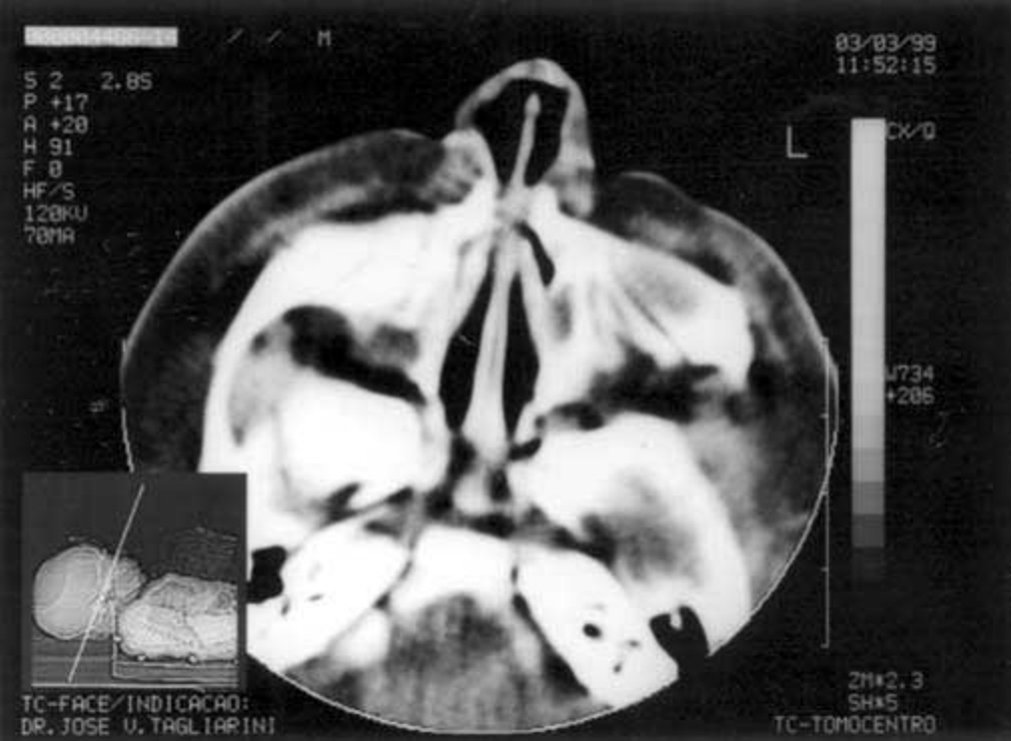
Pyriform aperture stenosis.

**Figure 3:**
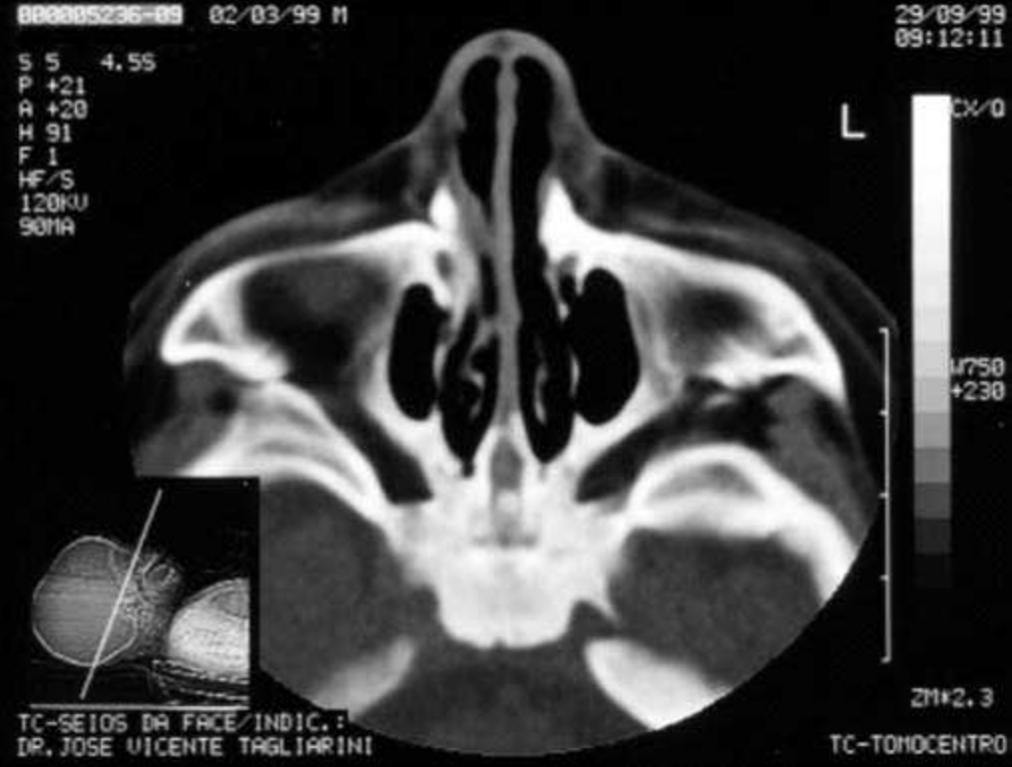
Follow-up exam 7 months after surgery.

**Figure 4:**
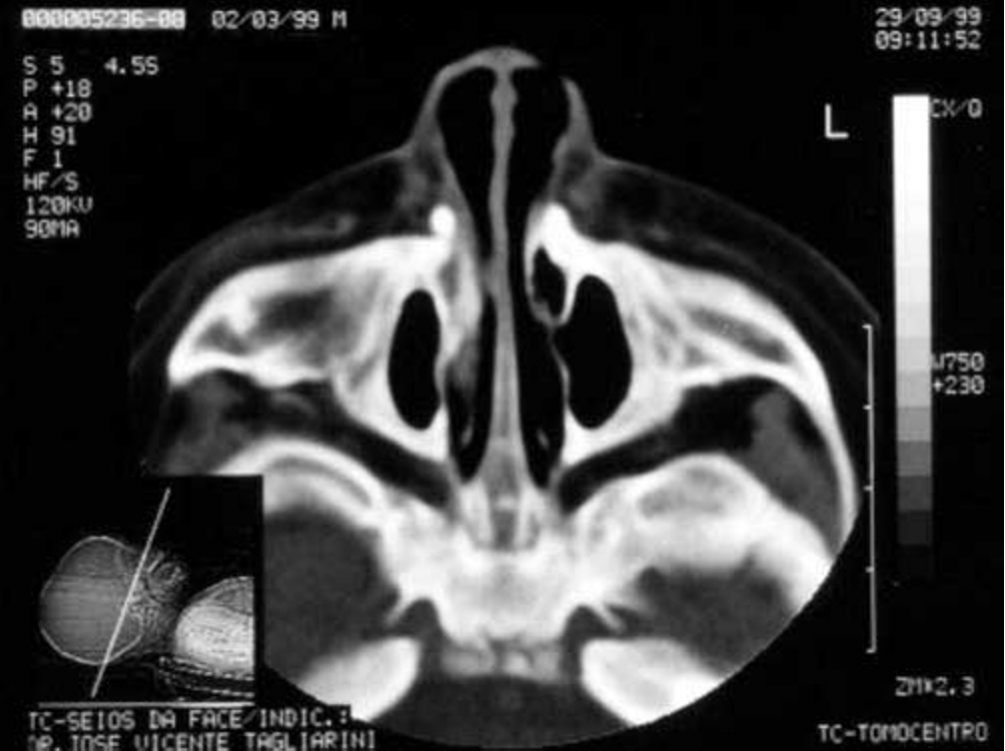
Follow-up exam 7 months after surgery.

**Figure 5:**
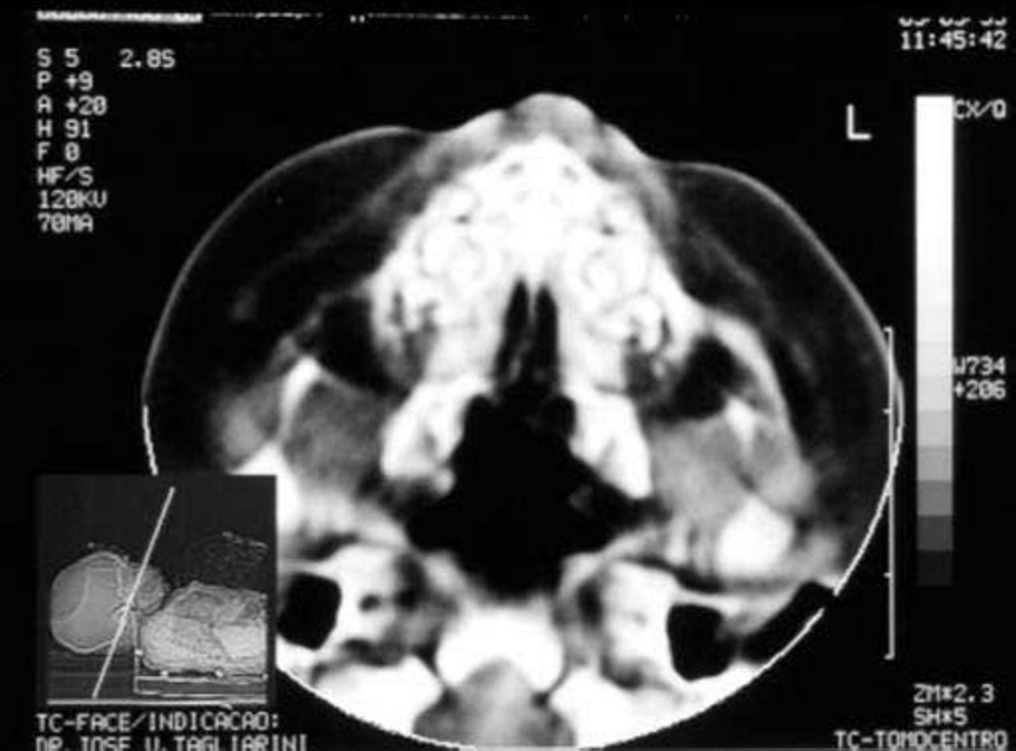
Evidence of central incisors.

Term baby, normal Apgar, weight of 3500g, had presented dyspnea, cyanosis and episodes of apnea since delivery. During initial assessment conducted by the pediatrician, it was not possible to pass a tube for nasal aspiration, as routinely performed in nursery facilities. We detected marked nasal obstruction with periods of cyanosis and difficulty to breastfeed. In view of these data, we came up with the diagnostic hypothesis of choanal imperforation. We immediately requested ENT assessment, and significant respiratory distress was detected with periods of cyanosis and apnea. At anterior rhinoscopy, we observed bone narrowing of nasal fossa on the anterior third and impossibility to introduce the polyethylene tube number 5 measuring 2mm diameter beyond the initial 1cm of both nasal fossae. Nasal CT scan demonstrated presence of marked congenital stenosis in both pyriform apertures (Figures 1 and 2). Owing to the picture of respiratory failure and inability the child had to feed, the physician indicated immediate surgery to correct stenosis of pyriform aperture. The surgery was conducted using sublabial approach. After exposure of the bone stenosis area, we worn out the area by using a micromotor and burs to correct pyriform stenosis. Bone wear was carefully made not to damage naso-lachrymal pathways and recovering mucosa of nasal fossae. After bone correction, we conducted longitudinal incision of mucosa on the anterior third of the nasal fossa floor to widen the lumen of the nasal fossa and adjust mucosa recovering layer to the new diameter of nasal fossa. We did not use nasal packing but rather a sylastic nasal splint to prevent formation of adherences. On the 5th postoperative day, we removed the nasal splint and used weekly dressings with good evolution. The child presented improved nasal breathing, with consequent weight gain and she presents normal development at the age of 3 years, with physical and facial growth within the normal range. Deciduous teeth grew without abnormalities. We conducted control paranasal sinuses CT scan at seven months and two years to demonstrate good development of nasal fossae even though she still had partial narrowing of the middle third (Figures 3 and 4), in addition to presence of dental germens of central incisors and absence of associated malformation (Figure 5). The child was followed up until the age of 3 years, when this case was reported.

## DISCUSSION

CSPA is the cause of neonatal nasal obstruction that may take to nasal obstruction, ranging from mild to severe. In the case reported here, the patient presented dyspnea, cyanosis and apnea. Given that newborns are preferably nasal breathers, any form of severe nasal obstruction may cause respiratory failure and its recognition may prevent asphyxia^1^, ^8^, ^11^. These signs and symptoms occur in bilateral choana atresia, an most well-known etiology, and therefore, it could be the etiology assumed by the neonatologist^1^, ^8^, ^11^. Nasal fossa examination in this patient showed that the aspiration tube did not progress more than 1cm into the nasal fossae. This observation led to a more detailed examination of the anterior third of the nasal fossae. Anterior rhinoscopy of the newborn may be difficult to be performed owing to the small size of structures^11^. The exam showed stenosis of nasal fossa caused by submucous bone protrusion in pyriform aperture. Similarly to previous reports, nasal fossae lumen was below 2mm^11^. CT scan allowed confirmation of the diagnosis and normal anatomy of choanae^11^, and axial sections should always be made from the palate to the roof of the orbit^11^.

Mild cases of CSPA, after diagnosis, can be treated more conservatively, with humidification and topical nasal decongestants^1,^^8^, ^11^. Severe cases, such as the one presented, require surgical approach^1^, ^2^, ^3^, ^4^, ^5^, ^6^, ^7^, ^8^, ^11^. Surgical repair of CSPA comprises resection of inferior margin of the bone in anterior nasal aperture, which can be made by two accesses. Transnasal access has been used in adults and it is technically more difficult in neonate noses. Sublabial approach using microscope, as performed in this case, allows excellent visualization and preservation of nasal mucosa. Nasolachrymal ducts should be dissected and preserved. We should be careful at the level of the nose floor to prevent damage of dental germens^1^, ^11^. The aperture bone should be worn out sufficiently to widen pyriform aperture and allow passage through nasal fossa lumen of an endotracheal tube of 3.5mm^11^. We recommend the use of a stent for a period of 1 to 4 weeks and in this patient we used a splint formed by sylastic and fixed to the nasal septum with nylon stitches^1^, ^8^, ^11^. We made a mucosa incision on the floor of the fossae to allow adaptation of the mucosa to the new nasal fossa dimension. Potential surgical complications include damage to dental germens and lachrymal duct, which can be prevented by careful surgical technique. During follow-up of our patient, we did not detect hypoplastic development of the nose on the mid third of the face^11^.

## CONCLUSION

CSPA, even though not very frequent, is an important cause of neonatal nasal obstruction. A respiratory pattern of cyclic cyanosis relieved by cry may be found and if not properly treated, it may lead the baby to asphyxia. Diagnosis seems to be conceptually easy because it is a deformity in the anterior portion of the nasal fossae, which is theoretically accessible to physical examination. However, it may be difficult owing to size of structures, and the final conclusion is reached only using CT scan. Appropriate treatment in cases of severe obstruction allows preservation of patients' lives.
